# Comparison of antibody responses before and after booster doses with the Pfizer-BioNTech or Oxford–AstraZeneca vaccines in healthcare workers in Thailand

**DOI:** 10.1016/j.jvacx.2023.100277

**Published:** 2023-02-20

**Authors:** Uraporn Phumisantiphong, Sirichan Chunhakan, Anan Manomaipiboon, Jakravoot Maneerit, Pornchai Dechanuwong, Thananda Trakarnvanich, Wadakorn Oajaum, Wilawan Chan-in

**Affiliations:** a Department of Clinical Pathology, Faculty of Medicine Vajira Hospital, Navamindradhiraj University, Bangkok, Thailand; b Department of Surgery, Faculty of Medicine Vajira Hospital, Navamindradhiraj University, Bangkok, Thailand; c Department of Orthopedics, Faculty of Medicine Vajira Hospital, Navamindradhiraj University, Bangkok, Thailand; d Department of Medicine, Faculty of Medicine Vajira Hospital, Navamindradhiraj University, Bangkok, Thailand; e Renal Division, Department of Medicine, Vajira Hospital, Navamindradhiraj University, Bangkok, Thailand; f Department of Central Laboratory, Faculty of Medicine Vajira Hospital, Navamindradhiraj University, Bangkok, Thailand

**Keywords:** COVID-19, Anti-SARS-CoV-2 S, Vaccine, Pfizer-BioNTech, Oxford–AstraZeneca

## Abstract

The severe acute respiratory syndrome 2 (SARS-CoV-2) has spread rapidly worldwide, not only causing significant morbidity and mortality but also dramatically increasing health care spending. To manage this in Thailand, healthcare workers first received two doses of the CoronaVac vaccine followed by a booster vaccine with either BNT162b2 vaccine (Pfizer-BioNTech; PZ) or ChAdOx1 nCoV-19 vaccine (Oxford–AstraZeneca; AZ). Given that the difference in anti-SARS-CoV-2 levels following vaccination may vary depending on the vaccine and on demographic characteristics, we measured the antibody response after the second CoronaVac dose and after the booster with either the PZ or AZ vaccine. Our results in 473 healthcare workers show that the variation in antibody response to the full CoronaVac dose depends on demographic characteristics such as age, gender, body mass index, and underlying disease. After receiving a booster dose, anti-SARS-CoV-2 levels were significantly higher in participants who received the PZ vaccine than in people who received the AZ vaccine. Overall, however, receiving a booster dose of either the PZ or AZ vaccine promoted strong antibody responses, even in the old and those with obesity or diabetes mellitus. In conclusion, our results support the use of a booster vaccination program after full vaccination with the CoronaVac vaccine. This approach effectively enhances immunity against SARS-CoV-2, especially in clinically vulnerable groups and healthcare workers.

## Highlight

• Variation in antibody response to the full CoronaVac dose depends on age, gender, body mass index, and underlying disease, consistent with earlier research.
• After receiving a booster dose, antibody levels were significantly higher in participants who received the PZ vaccine than in those who received the AZ vaccine.
• Receiving either PZ or AZ booster dose promoted strong antibody responses, even in the old and those with obesity or diabetes mellitus.

## Introduction

Severe acute respiratory syndrome coronavirus 2 (SARS-CoV-2) caused the coronavirus disease 2019 (COVID-19), which started in Wuhan, China, in late December 2019. Since then, COVID-19 spread throughout China [1], [2] and to several countries worldwide, leading the World Health Organization (WHO) to classify the outbreak a pandemic on March 11, 2020 [3]. The COVID-19 pandemic has had dramatic economic and social effects in each affected country, with disruptions to services, workplaces, schools and universities, and trade and tourism. The disease has been notable for requiring patients to be admitted to hospitals with greater resource requirements.

In Thailand, from 3 January 2020 to 13 January 2023, there have been 4,724,916 confirmed cases of COVID-19 with 33,727 deaths, reported to WHO. [3]. The Thai Ministry of Health have produced various guidelines and recommendations to prevent and reduce the spread of infection, including proactive screening to detect infection in at-risk populations or to confirm the source of an outbreak, and a vaccination program against COVID-19 that has targeted healthcare workers and at-risk populations. Vaccines are effective for dealing with epidemics [4] by helping to strengthen the immunity of the vaccinated and preventing severe disease. Besides enhancing personal immunity, a sufficiently effective COVID-19 vaccination program will lead to herd immunity and further contribute to preventing spread to the unvaccinated or non-immune [5]. Therefore, COVID-19 vaccination may prove essential for controlling the escalating epidemic in Thailand.

Most healthcare workers in Thailand, as a priority group for COVID-19 vaccination, have already received a full two-dose regimen of the CoronaVac (Sinovac) inactivated vaccine in April-May 2021 [6]. Since receiving this vaccination, the Delta variant was the predominant Thailand circulating SARS-CoV-2 strain displacing the Alpha variant, even in some vaccinated individuals [7]. In December 2021, the first case of the Omicron variant in Thailand was confirmed, and spread rapidly became the dominant SARS-CoV-2 variant.

The Thai Ministry of Health recommends a third booster vaccine for healthcare workers who received 2 doses of CoronaVac to provide higher protection against COVID-19. Since August 2021, three booster vaccines are available: ChAdOx1 nCoV-19 (Oxford–AstraZeneca; AZ), a viral vector vaccine [8], BNT162b2 (Pfizer-BioNTech; PZ) [9], and mRNA-1273 (Moderna), messenger RNA vaccines [10]. Given that healthcare workers treating patients with, under investigation for, COVID-19 are key workers, we must know the effect of the booster vaccination program on the antibody response in these personnel.

In this study, we aimed to compare antibody levels against SARS-CoV-2 after the second CoronaVac vaccine and the third doses of the PZ and AZ vaccine in healthcare workers, together with their demographic characteristics affecting antibody levels after vaccination.

## Materials and methods

### Study design, participants, and data collection

This prospective study was conducted at the Faculty of Medicine, Vajira Hospital, Navamindradhiraj University (Bangkok, Thailand) between March 2021 and September 2021. We invited 500 healthcare workers who had received two doses of CoronaVac vaccine (Sinovac Life Sciences, Beijing, China) and a booster dose with either the AZ vaccine (AstraZeneca-Oxford University, Oxford, UK) or the PZ vaccine (Pfizer-BioNTech, NY, USA), or without a booster dose. Data were collected by electronic questionnaire, included demographic characteristics, comorbidities, and clinical questions about COVID-19. The participants were asked about their history of underlying medical conditions, including diabetes mellitus (DM), hypertension, hyperlipidemia, bleeding disorder, asthma, chronic obstructive pulmonary disease (COPD), cardiovascular disease, cancer, and autoimmune disease. We excluded anyone unwilling to provide information or with a COVID-19 diagnosis by polymerase chain reaction within the last 3 months. Participants received a booster dose of the PZ or AZ vaccine between 92 and 104 days after the second CoronaVac dose. The Ethics Committee of the Faculty of Medicine, Vajira Hospital, Navamindradhiraj University (Ref no. COA-099/2021) approved this study and participants signed an informed consent form on recruitment.

## Blood collection and SARS-CoV-2 antibody quantification

We collected two blood samples from all participants by standard venipuncture procedure, one at 4–5 weeks after the second dose of the CoronaVac vaccine and one at 4–5 weeks after the PZ or AZ vaccine booster.

Antibody quantification was then performed by electrochemiluminescence, using the Elecsys Anti-SARS-CoV-2 S immunoassay (Roche Diagnostics, Mannheim, Germany). Serum samples were tested and analyzed on a Cobas pro e801 analyzer (Roche Diagnostics Rotkreuz, Switzerland) at the Central Laboratory, Faculty of Medicine Vajira Hospital, Navamindradhiraj University. They reported the antibody responses against the receptor-binding domain of the S1 subunit of the spike protein, considering values ≥ 0.8 U/mL as positive and values < 0.8 U/mL as negative, according to the manufacturer’s cut–off point. Although the instrument has a stated measurement range of 0.4–250 U/mL, it can be expanded by automatic dilution when antibody levels exceed the upper limit. In this study, we convert the anti-SARS-CoV-2 S measurement unit from units per milliliter into the WHO standard unit, binding antibody units (BAU)/mL, by multiplying the reported value (U/mL) by 1.029 [11].

### Statistical analysis

Data were imported into ​​GraphPad Prism 5.0 software (San Diego, CA) for analysis. Descriptive data are shown as median and interquartile range. Mann-Whitney U and Kruskal-Wallis H tests were used to determine significant difference among groups. A p-values < 0.05 was considered statistically significant.

## Results

### Demographic data of included individuals

**Fig. 1:**
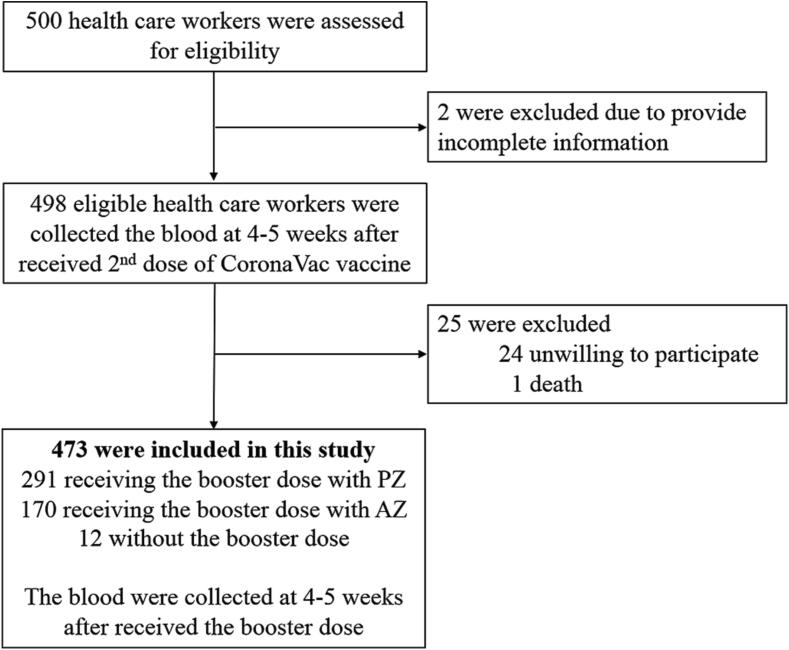
Assessment for eligibility.

In total, 473 healthcare workers had their antibody levels against SARS-CoV-2 S analyzed after both the second dose of the CoronaVac vaccine and the booster dose with either the PZ (N = 291) or the AZ (N = 170) vaccine, and without the booster dose (N = 12) (Fig. 1). Table 1 summarizes the population characteristics (age range, 20–60 years; females, 84.1 %). Many had a normal body mass index (BMI) (44.8 %), according to the Asian cut–off criteria [12], and most (65.1 %) had no underlying diseases. The most common comorbidities in the remaining participants were diabetes mellitus (DM), hyperlipidemia, and hypertension.

**Table 1:** Demographic data of included individuals.

	N (%)
All	473 (100 %)
Age (years)	
20–29	129 (27.3 %)
30–39	124 (26.2 %)
40–49	131(27.7 %)
50–60	89 (18.8 %)
Gender	
Male	75 (15.9 %)
Female	398 (84.1 %)
BMI (Asian criteria BMI cut-off)	
<18.5 (underweight)	46 (9.7 %)
18.5–22.9 (healthy weight)	212 (44.8 %)
23–24.9 (overweight)	82 (17.4 %)
25–29.9 (pre-obese)	89 (18.8 %)
>30 (obese)	44 (9.3 %)
Blood group	
A	106 (22.4 %)
B	146 (30.9 %)
AB	40 (8.5 %)
O	179 (37.8 %)
Unknown	2 (0.4 %)
Underlying diseases	
Absent	308 (65.1 %)
Present	165 (34.9 %)
• Diabetes mellitus (DM)	20 (4.2 %)
• Hyperlipidemia	57 (12 %)
• High blood pressure (BP)	34 (7.2 %)
• Other	80 (16.9 %)
Booster (3rd) dose vaccine type	
Without SARS-CoV-2 infection history	456 (96.4 %)
• PZ	283 (59.8 %)
• AZ	167 (35.3 %)
• Without booster	6 (1.3 %)
With SARS-CoV-2 infection history	17 (3.6 %)
• PZ	8 (1.7 %)
• AZ	3 (0.6 %)
• Without booster	6 (1.3 %)

N = number.

## Anti-SARS-CoV-2 S level after the full CoronaVac vaccine

**Fig. 2:**
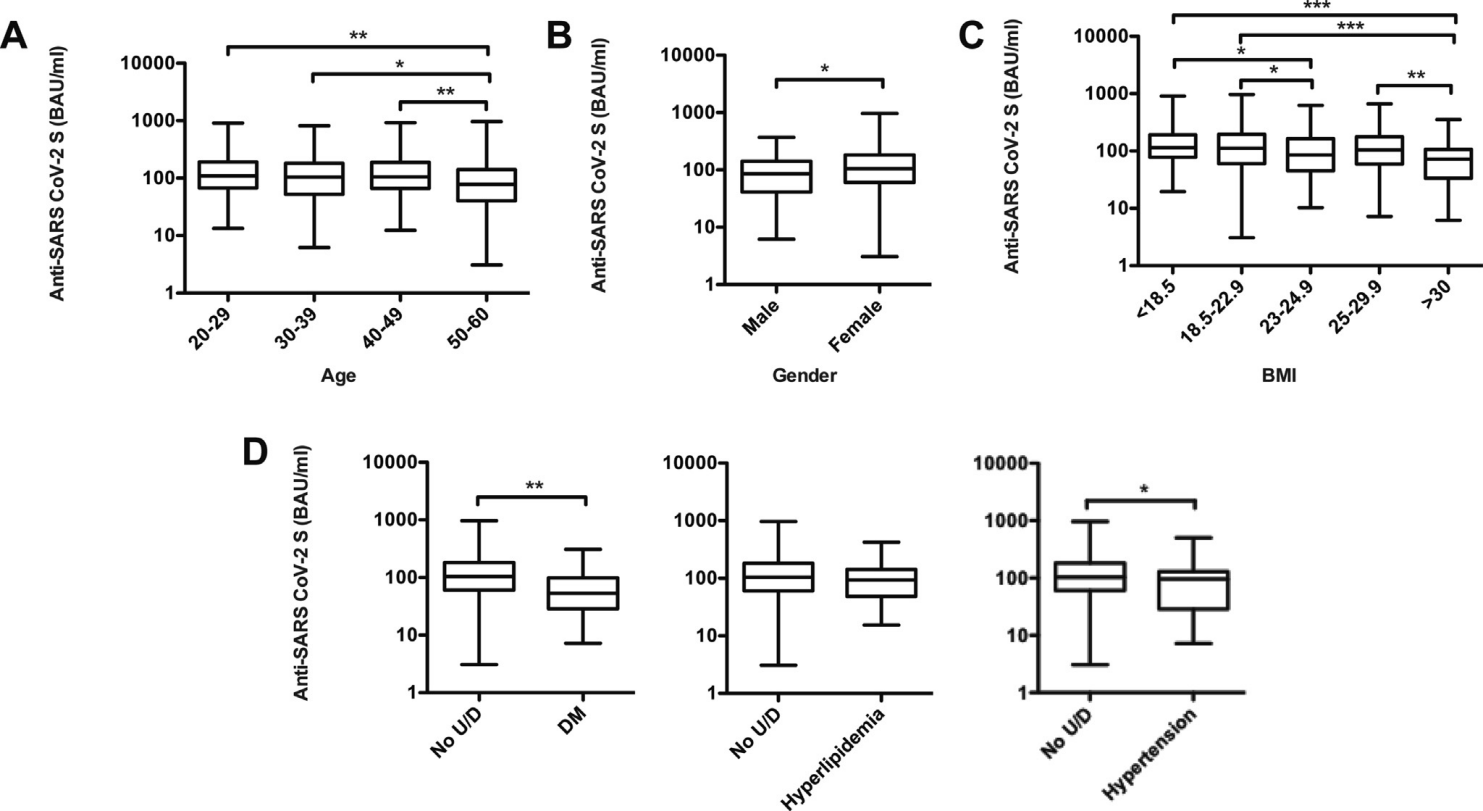
**Anti-SARS-CoV-2 S levels after the second CoronaVac vaccine.** The graph shows the median (middle line) and interquartile range of anti-SARS-CoV-2 S level (BAU/mL). We used Mann-Whitney U and Kruskal-Wallis H tests for statistical comparison, considering p < 0.05 as statistically significant (*p < 0.05, **p < 0.01, ***p < 0.001).

There was a significant difference in the antibody response after second CoronaVac dose between the different age categories (p = 0.0172) (Fig. 2A). The level of antibody were significantly lower in participants aged 50–60 years (median 76.00 BAU/mL; interquartile range 39.00–136.50 BAU/mL) compared to those aged 20–29 years (106.50 BAU/mL; 65.26–185.50 BAU/mL; p = 0.0030), 30–39 years (101.00 BAU/mL; 51.00–176.50 BAU/mL; p = 0.0233), and 40–49 years (76.00 BAU/mL; 64.25–183.30 BAU/mL; p = 0.0064) (Fig. 2A). By sex, antibody responses were significantly higher in females (105.00 BAU/mL; 60.71–183.2 BAU/mL) compared to males (85.41 BAU/mL; 41.16–142.00 BAU/mL; p = 0.0113) (Fig. 2B). According to their BMI, obese participants (BMI > 30) had significantly lower antibody responses (70.00 BAU/mL; 32.50–104.00 BAU/mL) compared to those with BMIs of < 18.5 kg/m^2^ (111.00 BAU/mL; 75.50–186.50 BAU/mL; p = 0.0003), 18.5–22.9 kg/m^2^ (109.00 BAU/mL; 58.50–190.50 BAU/mL; p = 0.0002), and 25–29.9 kg/m^2^ (101.00 BAU/mL; 57.50–172.00 BAU/mL; P = 0.0032) (Fig. 2C). A Kruskal-Wallis test showed that there was a significant difference in antibody level between the different BMI categories (P = 0.0011) (Fig. 2C). Participants with underlying DM (52.00 BAU/mL; 28.00–96.00 BAU/mL) and hypertension (96.21 BAU/mL; 28.81–130.20 BAU/mL) also showed significantly lower antibody responses compared to those without underlying diseases (101.00 BAU/mL; 59.00–178.00 BAU/mL; p = 0.054 and p = 0.0363 respectively) (Fig. 2D), but we found no significant differences for hyperlipidemia in similar comparisons. Furthermore, we found no significant difference in antibody responses by blood group (S1 Fig).

## Anti-SARS-CoV-2 S level and fold-change in level per individual after receiving the PZ or AZ booster

**Fig. 3:**
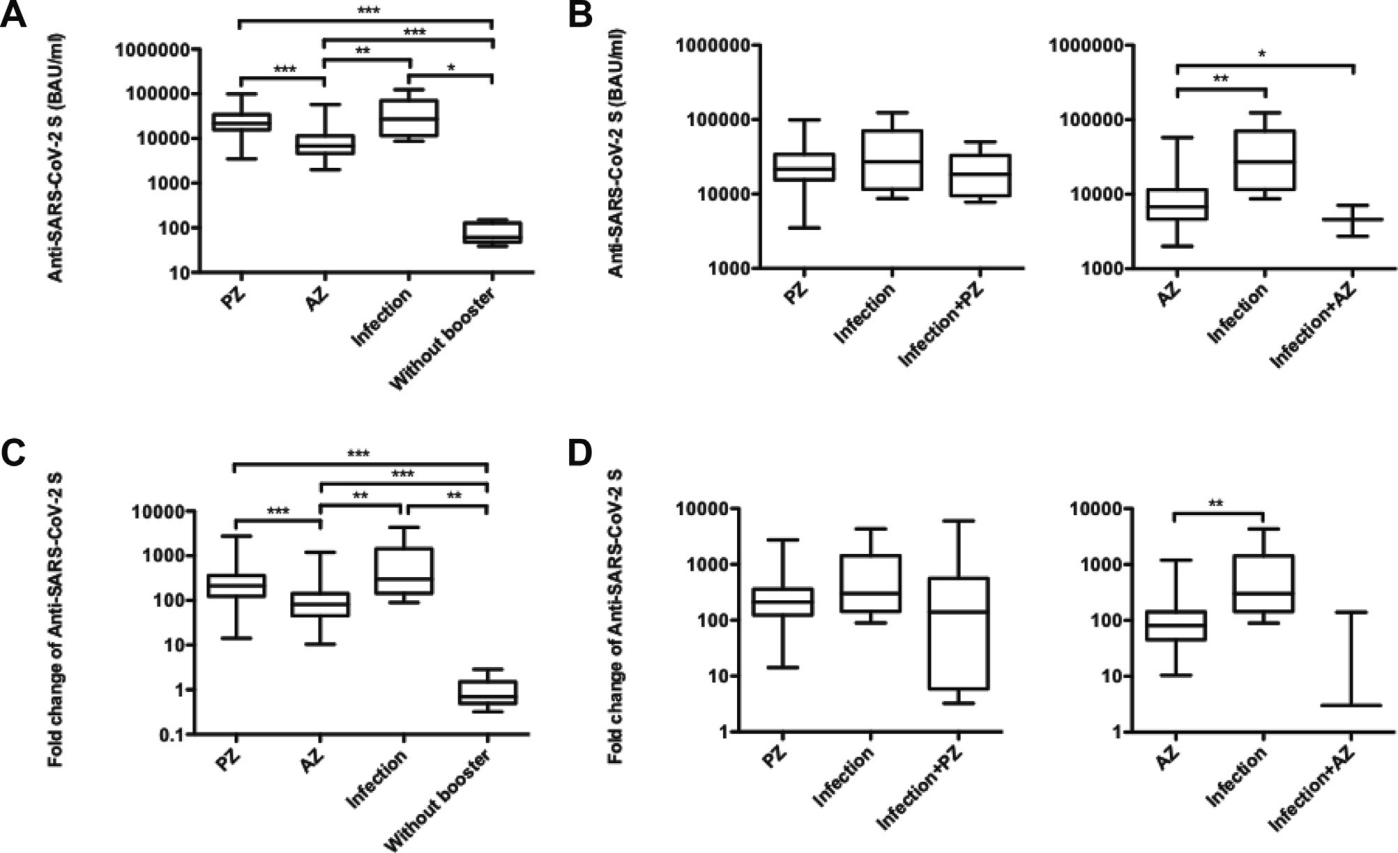
**Anti-SARS-CoV-2 S levels and fold-changes in levels after the with PZ or AZ booster dose per individual.** The graph shows the median (middle line) and interquartile range of anti-SARS-CoV-2 S levels (BAU/ml) (A, B) with the fold-change in that level (C, D). We used Mann-Whitney U and Kruskal-Wallis H tests for statistical comparison, considering p < 0.05 as statistically significant (*p < 0.05, **p < 0.01, ***p < 0.001).

Antibody responses in participants who received the PZ booster (21,457 BAU/mL; 15,571–34,266 BAU/mL) were significantly higher than in participants who received the AZ booster (6,763 BAU/mL; 4,626–11,525 BAU/mL; p < 0.0001) (Fig. 3A). However, participants without a booster dose (60.97 BAU/mL; 47.95–127.9 BAU/mL) had significantly lower antibody responses than participants receiving either the PZ booster (21,457 BAU/mL; 15,571–34,266 BAU/mL; p < 0.0001) or AZ booster (6,763 BAU/mL; 4,626–11,525 BAU/mL; p < 0.0001) (Fig. 3A). A Kruskal-Wallis test showed that there was a significant difference in antibody level between the different boosters (P < 0.0001) (Fig. 3A). Additionally, participants who acquire natural SARS-CoV-2 infection after they received the full dose CoronaVac dose had significantly higher antibody responses (27,153 BAU/mL; 11,590–70,521 BAU/mL) than participants who received the AZ booster dose (6,763 BAU/mL; 4,626–11,525 BAU/mL; p = 0.0012) (Fig. 3A). Among these patients with natural infection after receiving the full CoronaVac vaccine, we found no significant differences in antibody responses between those who then did not receive a booster (27,153 BAU/mL; 11,590–70,521 BAU/mL) and those who later received a booster dose with PZ (18,412 BAU/mL; 9,466–33,004 BAU/mL) or AZ (4,575 BAU/mL; 2,734–7,115 BAU/mL) (Fig. 3B).

To compare the change in antibody response from before to after receiving the booster dose, we calculated the fold-change in the anti-SARS-CoV-2 S level per individual. This involved dividing the anti-SARS-CoV-2 S level before the booster with that after the booster. There was a significant difference in the fold-changes of antibody response between the different boosters (p < 0.0001) (Fig. 3C). Participants who received the PZ booster (211.40; 123.70–359.60) had significantly higher fold-changes than those who received the AZ booster (81.03; 45.19–131.40; p < 0.0001) (Fig. 3C), but either booster produced significantly higher fold-changes (211.40; 123.70–359.60 and 81.03; 45.19–131.40, respectively) than not giving a booster (0.70; 0.49–1.50; p < 0.0001 and < 0.0001, respectively). Natural infection after the full CoronaVac dose produced the highest fold-changes (301.00; 144.10–1,430.00; p = 0.0029 compared to the AZ booster) (Fig. 3C); when this group subsequently received a booster, the fold-change in anti-SARS-CoV-2 S level per individual did not change significantly (PZ: 139.70; 5.89–561.20, AZ: 3.00; 3.00–140.00) (Fig. 3D).

## Association between demographic data and fold-change in anti-SARS-CoV-2 S level after receiving a booster dose

**Fig. 4:**
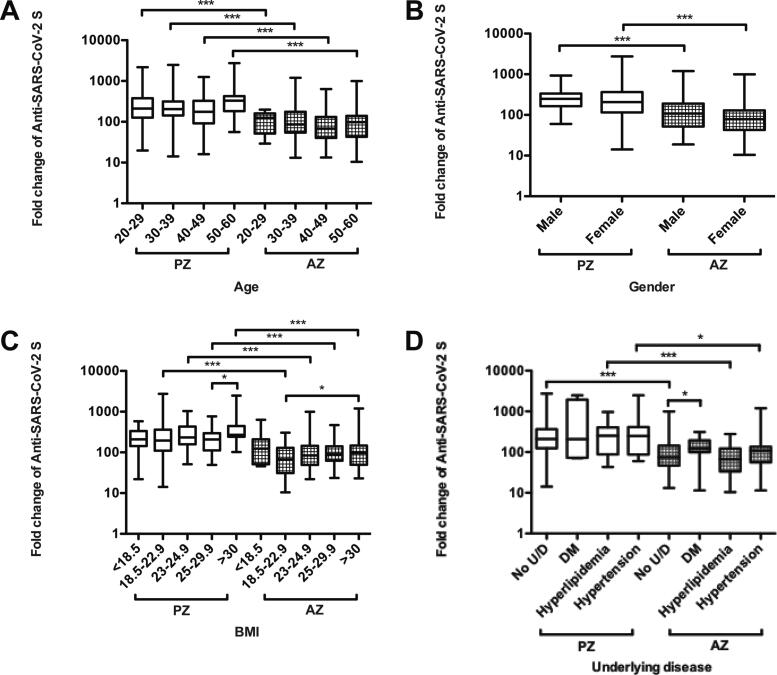
**Association between demographic data and fold-change in anti-SARS-CoV-2 S level after the booster dose.** The graph shows the median (middle line) and interquartile range of the fold-change in anti-SARS-CoV-2 S level. We used Mann-Whitney U and Kruskal-Wallis H tests for comparison, considering p < 0.05 as statistically significant (*p < 0.05, **p < 0.01, ***p < 0.001).

Participants in all age groups receiving the PZ booster (20–29 years: 211.80; 125.90–378.10, 30–39 years: 204.50; 142.40–314.20, 40–49 years: 174.70; 90.79–323.80, 50–60 years: 330.80; 182.40–424.70) showed significantly higher fold-changes in anti-SARS-CoV-2 S levels compared to those receiving the AZ booster (20–29 years: 123.30; 51.55–160.50; p = 0.0007, 30–39 years: 85.83; 54.23–176.00; p < 0.0001, 40–49 years: 67.15; 40.04–130.80; p < 0.0001, 50–60 years: 98.41; 42.68–140.00; p < 0.0001) (Fig. 4A). However, no statistically significant differences existed when comparing between age groups that received the same type of booster (PZ or AZ) (Fig. 4A).

By sex, males (248.00; 164.40–355.10) and females (205.90; 114.90–363.30) who received the PZ booster showed higher fold-changes in anti-SARS-CoV-2 levels than males (108.80; 51.97–189.70; p < 0.0001) and females (78.59; 42.67–129.70, p < 0.0001) who received the AZ vaccine (Fig. 4B).

The fold-changes in anti-SARS-CoV-2 level were significantly higher for participants who received the PZ booster at almost all BMI ranges (18.5–22.9 kg/m^2^: 196.20; 110.10–360.50, 23–24.9 kg/m^2^: 233.30; 157.70–431.00, 25–29.9 kg/m^2^: 207.50; 111.80–297.20, >30 kg/m^2^: 269.30; 242.50–443.80) when compared to those who received the AZ booster (18.5–22.9 kg/m^2^: 67.18; 30.97–129.7; p < 0.0001, 23–24.9 kg/m^2^: 84.25; 49.35–145.50; p < 0.0001, 25–29.9 kg/m^2^: 89.92; 62.49–140.60; p < 0.0001, >30 kg/m^2^: 96.34; 49.72–147.30; p < 0.0001) (Fig. 4C). Among those who received the PZ booster, the fold-change was significantly higher for the BMI group > 30 kg/m^2^ (269.30; 242.50–443.80) than for BMI group 25–29.5 kg/m^2^ (207.50; 111.80–297.20; p = 0.016). After the AZ booster, the fold-change was significantly different between BMI group 18.5–22.9 kg/m^2^ (67.18; 30.97–129.7) and > 30 kg/m^2^ (96.34; 49.72–147.30; p = 0.0227) (Fig. 4C).

We found no significant difference in the fold-change of anti-SARS-CoV-2 S between the PZ and AZ groups among participants with underlying DM (Fig. 4D). However, participants with DM who received the AZ booster showed higher fold-changes (123.10; 99.39–194.70) than those without underlying disease who received the AZ booster (74.78; 46.44–143.80; p = 0.0304) (Fig. 4D). While participants with underlying hyperlipidemia and hypertension who received PZ booster (252.70; 88.30–404.40 and 247.60; 87.68–412.40 respectively) showed significantly higher fold-change compared to those received AZ booster (67.03; 33.62–123.00; p < 0.0001 and 108.50; 56.16–135.10; p = 0.0225).

## Discussion

In this study, we investigated the anti-SARS-CoV-2 antibody response after receiving both the full CoronaVac dose and a PZ or AZ booster dose in healthcare workers who treat patients with, or under investigation for, COVID-19. Three broad conclusions are possible. First, we showed that variation in antibody response to the CoronaVac vaccine depends on factors such as age, gender, BMI, and underlying disease (notably DM). Second, people who received the full CoronaVac dose followed by the PZ booster have higher antibody responses than people who received the AZ booster. However, among people who acquire natural infection after the second CoronaVac dose, antibody responses are similar irrespective of whether they subsequently receive a PZ or AZ booster. Third, both the PZ and AZ boosters promote strong antibody responses in all participants, including the clinically vulnerable (i.e., old age, obesity, and DM).

Our findings show that variation in the SARS-CoV-2 S antibody response after receiving the full CoronaVac dose depended on demographic characteristics, being lowest in older age, male sex, obesity, and DM. These lower immune responses are consistent with reports showing that older age [13], [14], [15], male sex [13], [14], [15], [16], obesity [14], [15], and DM [13], [14], [15] confer high-risk for severe disease and hospitalization during SARS-CoV-2 infection. However, a recent study found that no significant relationship existed between sex and the hospitalization rate for COVID-19 [17].

After receiving either booster vaccine, anti-SARS-CoV-2 S levels increased over 10-fold in all participants compared to the levels after receiving the full CoronaVac dose. Previous studies reported higher level of anti-SARS-CoV-2 S levels in individuals vaccinated with PZ compared to those vaccinated with AZ [18], [19]. There is a possibility that higher antibody levels are associated with greater protection against SARS-CoV-2 [19]. However, both approaches to booster dosing, either using mRNA or viral vector, should provide protection against COVID-19 for a population that has received inactivated vaccines (e.g., CoronaVac) by increasing anti-SARS-CoV-2 S levels. Consistent with our findings, recent studies show that administration of the third dose of vaccine with PZ [20], [21], [22] or AZ [21], [22] after receiving the full CoronaVac dose induced a significant increase in anti-SARS-CoV-2 level, and the highest antibody concentrations are observed after boosted mRNA vaccine [21], [22]. The increasing anti-SARS-CoV-2 levels after receiving the third dose of the vaccine with PZ or AZ are binding and neutralizing antibodies against both delta and omicron variants, which could improve protection against infection [21], [22].

Among participants who acquired natural infection after the second dose of CoronaVac, we observed a significant difference in the antibody response between those who received an AZ booster and those who did not. On the other hand, no significant difference was detected in the antibody response between participants who received a PZ booster. Nevertheless, due to the limited sample size in this group, it is not feasible to reach a conclusion regarding the necessity of a booster dose for this population.

Despite low anti-SARS-CoV-2 S levels after the second CoronaVac dose being associated with older age, obesity, and DM, we observed high fold-changes in anti-SARS-CoV-2 levels after participants received a booster dose with the PZ or AZ vaccine, irrespective of age, gender, BMI, and underlying disease. Therefore, the booster dose should be considered essential for these groups with higher risk of severe disease and hospitalization.

In summary, our results support the implementation of a booster vaccination program with currently available mRNA or viral vector vaccines in people who have previously received an inactivated vaccine (specifically CoronaVac). This approach leads to an increase in antibody levels, which is expected to be associated with protection against SARS-CoV-2, especially in high-risk groups, such as the clinically vulnerable and healthcare workers involved in treating COVID-19. Our study limitation is the lack of neutralizing antibody testing. However, the correlation between the anti-SARS-CoV-2 S level and neutralizing antibodies has been described in literature [23].

## Ethical approval

The study protocol was approved by the Institutional Review Board of the Faculty of Medicine Vajira Hospital, Navamindradhiraj University (Ref no. COA-099/2021).

## Funding

This study was supported by Navamindradhiraj University Research Fund (Grant no. 85/2564).

## Declaration of Competing Interest

The authors declare that they have no known competing financial interests or personal relationships that could have appeared to influence the work reported in this paper.

## Figures and Tables

**Supplementary figure 1:**
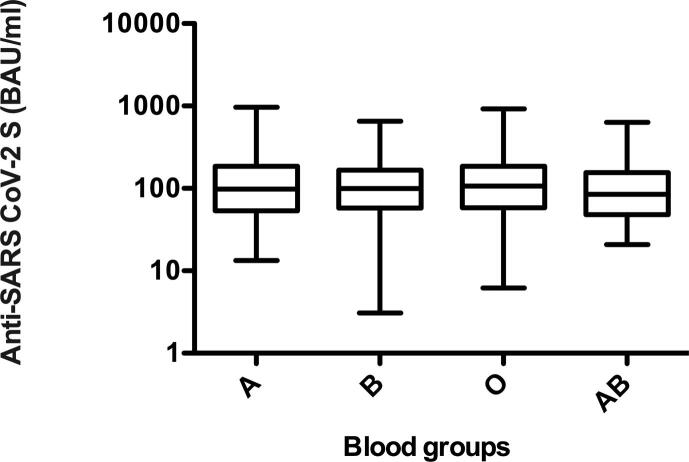
Anti-SARS-CoV-2 S level by blood group. The graph shows the median (middle line) and interquartile range of anti-SARS-CoV-2 S level (BAU/mL). We used Kruskal-Wallis H tests for comparison, considering p < 0.05 as statistically significant (*p < 0.05, **p < 0.01, ***p < 0.001).

## Data Availability

The data that has been used is confidential.
